# “Pleasantly confused” and missed opportunities for delirium recognition: A hospital record audit

**DOI:** 10.1016/j.ijnsa.2026.100575

**Published:** 2026-05-25

**Authors:** Kelly Marriott-Statham, Eleanor Brace, Seán Hambrook, Chrysta Bridge, Kasia Bail

**Affiliations:** a University of Canberra, Australian Capital Territory (ACT), Australia; b Canberra Health Services, Canberra Hospital, ACT, Australia; c ACT Health Directorate, ACT, Australia

**Keywords:** Delirium, Documentation, Medical record review, Nursing staff, Patient safety, Retrospective studies

## Abstract

**Background:**

Delirium is a serious hospital complication with long-lasting and life-threatening consequences and high healthcare costs. Despite decades of evidence, tools, and guidelines, delirium remains under-recognised and under-treated.

**Objective:**

To examine alignment between policy and practice for delirium risk identification, screening, and assessment and to identify the language used in documentation to describe cognitive observations and delirium.

**Design:**

A retrospective, cross-sectional clinical documentation audit using a single-day point prevalence study design.

**Setting(s):**

A tertiary metropolitan hospital in southeastern Australia where the clinical records were audited.

**Participants:**

A midnight census conducted in July 2021 identified 807 inpatient clinical records, of which 460 records met the eligibility criteria for this study.

**Methods:**

Clinical records were audited for delirium risk identification, screening, assessment, and language use in documentation referring to cognition. Descriptive analyses were used.

**Results:**

Among the 460 included records, risk factors for delirium were identified in 316 (69%). Of these at-risk patients, only 28/316 (9%) received subsequent screening, and 12/316 (4%) had further assessment, which resulted in 3/316 (1%) having a documented diagnosis of delirium. We also identified 331 documented words and phrases describing cognition. Diagnostic language, such as ‘delirium,’ appeared in only 3% of 331 documented words and phrases. Ambiguous language and euphemisms accounted for 22% of terms and included phrases such as ‘pleasantly confused,’ which dismissed the seriousness and urgency of delirium.

**Conclusions:**

Despite frequent risk identification, subsequent screening, assessment, and diagnostic naming of delirium was uncommon, creating missed opportunities for recognition and escalation. Ambiguous observations and euphemisms were documented by practitioners and diagnostic language omitted, rendering delirium invisible. Possible opportunities for increasing the detection and visibility of delirium in hospital practice include: nurses using advocacy language, such as ‘possible delirium’ and working diagnoses when screening scores are positive; electronic medical record prompts and alerts when information thresholds are met for patients; streamlined documentation to align everyday practice with delirium care pathway steps; and education alongside a change in institutional culture where cognitive care is seen as fundamental patient care.

**What is already known**

• Delirium is common in hospital settings, yet it persists as being under-recognised in routine care.
• Delirium care pathways follow a risk, screening, assessment, and planning/intervention workflow.
• Documentation about delirium in clinical records is usually vague or missing.

Alt-text: Unlabelled box dummy alt text

**What this paper adds**

• In a hospital-wide audit of clinical records, we found a cascade of failure across the delirium care pathway.
• Screening for delirium varied widely by admitting specialty.
• Despite 20 years of delirium research and implementation, healthcare practitioners in Australia favoured ambiguous words and phrases such as ‘confused,’ ‘pleasantly confused,’ and ‘agitated’ over ‘delirium’.

Alt-text: Unlabelled box dummy alt text

## 1 Background

Globally, delirium is known to be one of the most common and serious complications of hospitalisation. Studies estimate up to 34% of patients in hospital experience delirium (Koirala et al., 2020) with rates increasing to approximately half of older patients (Al Farsi et al., 2023), and around 80% of any-age mechanically ventilated patients in intensive care (Kumar et al., 2022). Researchers over several decades have generated robust evidence, tools, and guidelines for delirium care (Australian Commission on Safety and Quality in Health Care, 2021b; Crone et al., 2025; National Institute for Health and Care Excellence, 2023; Teodorczuk et al., 2023); however, delirium identification remains inadequate. Some researchers have suggested that between one and two thirds of delirium episodes are still being missed in hospital practice (Aggar et al., 2022; Al Farsi et al., 2023; Alhaidari and Matsis, 2022; Eagles et al., 2022; Titlestad et al., 2024), despite several repeated calls to action by leaders in delirium research and practice (Mazzola and Hascup, 2025; Teodorczuk et al., 2012; Thein et al., 2020; Traynor et al., 2025).

Delirium is a medical emergency, characterised by an acute disturbance in consciousness, attention, awareness, and cognition that develops quickly over hours or days (Australian Commission on Safety and Quality in Health Care, 2021b; Thom et al., 2019). Episodes are distressing for patients and families, which can have long-lasting psychological impacts (Aggar et al., 2022; Benn et al., 2025; Featherstone et al., 2021). The seriousness of delirium is further compounded by its known links with functional decline, dementia, discharge to long-term care, and death (Al Farsi et al., 2023; Dani et al., 2018; Gordon et al., 2024; Wu et al., 2021), alongside high economic burden, with the annual costs estimated in Australia of $8.8 billion (Caplan et al., 2020). Delirium is potentially reversible; but its successful management relies on timely identification and intervention by healthcare practitioners.

**Fig. 1:**
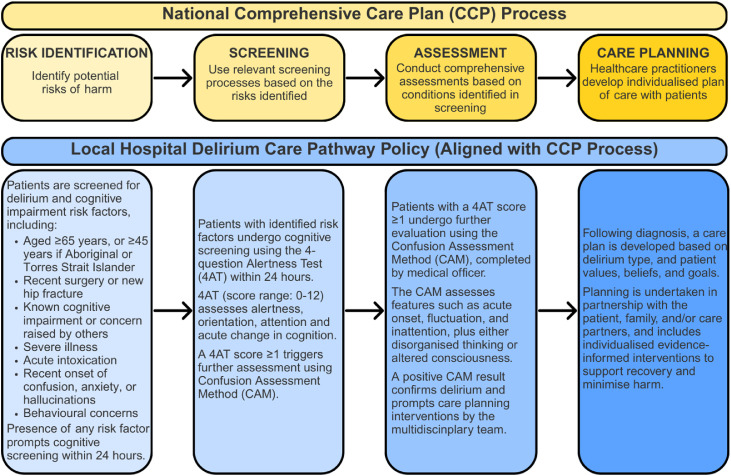
National Comprehensive Care Plan and alignment of local hospital policy pathway for delirium: risk identification, screening, assessment, and individual care planning.

Clinical record documentation shapes what is recognised and acted upon in hospital care, and routine screening contributes to the identification of delirium episodes (Cody et al., 2021; Liu et al., 2023). In Australia, delirium care is guided by the Delirium Clinical Care Standard and operationalised through the Comprehensive Care Plan, which directs healthcare practitioners to develop individualised plans that minimise patient harm and complications (Australian Commission on Safety and Quality in Health Care, 2021a, 2021b). The Comprehensive Care Plan aligns with the National Safety and Quality Health Service Standard, *Comprehensive Care*, and involves four steps: risk identification, screening, assessment, and care planning (Australian Commission on Safety and Quality in Health Care, 2021a) (Fig. 1). When followed, the Comprehensive Care Plan supports healthcare practitioners in the early identification and management of delirium and reflects evidence‑based practices outlined in the Delirium Clinical Care Standard (Australian Commission on Safety and Quality in Health Care, 2021b; Teodorczuk et al., 2023).

Routine screening is key to delirium identification and is commonly triggered by risk factors such as age, infection, recent surgery, cognitive impairment, or dementia (Australian Commission on Safety and Quality in Health Care, 2021b). Screening tools, such as the 4-item Alertness Test (4AT), have demonstrated high reliability and validity, yet their use remains inconsistent in practice (Alhaidari and Matsis, 2022; Lin et al., 2023; MacLullich et al., 2019; Tieges et al., 2020, 2021). Screening has been known to be omitted by healthcare practitioners who are unable to recognise cognitive impairment and in patients who are drowsy, have dementia, or do not speak English (Alhaidari and Matsis, 2022; Visser et al., 2024). When screening is embedded into routine nursing practice, possible delirium cases are more likely to be identified and acted upon (Australian Commission on Safety and Quality in Health Care, 2021b; Ragheb et al., 2023; Teodorczuk et al., 2023). Screening tools are sensitive enough to suggest possible delirium but are not diagnostic. Confirmation or exclusion of delirium is generally undertaken by a trained medical officer or psychiatrist using diagnostic criteria outlined in the Diagnostic and Statistical Manual of Mental Disorders or International Classification of Diseases (Lin et al., 2023; Shenkin et al., 2019). The Confusion Assessment Method, informed by these criteria, is widely used for its specificity and reliability but requires training and has been described by nurses as complex, subjective, and dependent on teamwork (Lin et al., 2023; Ragheb et al., 2023; Shenkin et al., 2019). These diagnostic challenges, combined with inconsistent screening and assessment, contribute to missed opportunities for intervention and management of delirium.

Beyond screening and assessment, documentation of confirmed delirium by healthcare practitioners is often ambiguous, with terms such as ‘confused’ used in place of ‘delirium’ (Chuen et al., 2021; Connolly et al., 2025; Puelle et al., 2015). For example, Chuen et al. (2021) found in their study only 31% of confirmed cases included ‘delirium’ documented on discharge summaries. In another study, diagnosis rates improved during a research intervention, but documentation did not, and terms like ‘confusion’ continued to replace ‘delirium’ (Welch and Jackson, 2018). Language choice shapes clinical responses, and it is known that diagnostic terms such as ‘delirium’ or ‘altered mental status’ predicted true delirium, whereas vague descriptors obscured urgency and reduced the likelihood of action (Puelle et al., 2015). Bowman et al. (2024) identified 78 different descriptors for delirium symptoms, many of which did not reflect the diagnostic criteria specified in the Diagnostic and Statistical Manual of Mental Disorders. Documentation, therefore, does more than record practice; it reflects institutional norms and workflow pressures. The way cognitive changes are described influences whether delirium becomes visible, recognised, and acted upon, or remains obscured within routine practice.

Despite clear delirium care pathways, validated tools, and national standards, delirium remains under-recognised and under-reported in hospitals. Few researchers have examined delirium care by following a care pathway, from risk identification to screening and assessment to diagnostic labelling and care planning. Further, the words and phrases used by healthcare practitioners in routine documentation following screening are unknown. Understanding how practitioners follow or deviate from delirium care pathways may help explain persistent under-recognition and inaction in hospital care. We aimed to examine adherence to a delirium care pathway and to characterise the language healthcare practitioners use in documentation following delirium screening in an Australian Hospital.

## 2 Methods

### 2.1 Study design and setting

We employed a retrospective, cross-sectional clinical documentation audit using a single-day point prevalence design to examine delirium screening, assessment, and documentation practices in a tertiary metropolitan hospital in southeastern Australia. A point prevalence approach was selected to enable examination of practice patterns without influencing healthcare practitioner behaviour. This approach enables inclusion of all hospital inpatients at a defined time point and has been used successfully in other studies of delirium documentation (Ankravs et al., 2020; Casey et al., 2019; Hosie et al., 2016; Koirala et al., 2020; Schiess et al., 2025). At the time this study was conducted, local hospital policy aligned delirium care with the national Delirium Clinical Care Standard and a Comprehensive Care Plan pathway, which guides sequential steps of risk identification, screening using the 4AT and further assessment using the Confusion Assessment Method (Fig. 1).

### 2.2 Participants (clinical records)

Clinical records were identified for inpatients listed in the midnight census on a date in late July 2021. Records were included if the patient was admitted at the census time and remained overnight. Records were excluded if the patient was aged ≤15 years, was admitted to mental health, justice health, alcohol and drug, paediatric, or maternity services. All eligible records at the census time were included.

### 2.3 Data variables, collection, and analysis

For each eligible record, data were extracted, de-identified, and entered in to a purpose-built, Microsoft Excel spreadsheet. The spreadsheet was piloted on 50 records prior to full data extraction. Following the pilot, the research team refined the spreadsheet to improve consistency, including confirming which cognition-related terms would be collected and adding additional items, such as the Glasgow Coma Scale. Variables were collected to align with each step of the Comprehensive Care Plan pathway and included demographics (age, sex/gender, Aboriginal or Torres Strait Islander identification), admitting specialty, length of stay, documented delirium risk identification (yes/no), screening using the 4AT (yes/no) and score, and assessment using the Confusion Assessment Method (yes/no) and score. Descriptive statistics were used to summarise data extracted.

The delirium screening and assessment variables included two tools used in the local delirium care pathway. The 4AT is a commonly used delirium screening instrument, recommended in many national healthcare standards (Lin et al., 2023; MacLullich et al., 2019). The 4AT takes approximately 2 min to complete, requires no training for use, and offers high reliability, sensitivity, and validity (Lin et al., 2023; MacLullich et al., 2019; Tieges et al., 2020, 2021). Higher scores indicate possible delirium or cognitive impairment, but the tool does not have sufficient specificity to confirm delirium (Lin et al., 2023; MacLullich et al., 2019; Tieges et al., 2020). Within this study, if a 4AT was initiated but not completed, it was recorded as not having been completed. The Confusion Assessment Method is informed by diagnostic criteria, has the specificity to confirm or exclude delirium, and is widely used due to its validity and reliability (Al Farsi et al., 2023; Lin et al., 2023). Specialised training is required for healthcare practitioners to use the Confusion Assessment Method, with positive delirium detection if there is presence of an acute onset and fluctuating course and inattention, as well as either disorganised thinking or an altered level of consciousness (Lin et al., 2023).

All eligible records were reviewed for completion of the 4AT, and, where completed, the next 24 hours of chronological documentation from all healthcare practitioners and disciplines (including medical, nursing, and allied health practitioners) in progress notes was reviewed for language related to cognition. Words and phrases indicating cognitive status or diagnoses (such as ‘confused,’ ‘agitated,’ ‘alert and orientated,’ ‘delirium’) were identified, counted, and recorded as key words. A term list was informed by literature, clinical knowledge and experience, and team discussion. During piloting and data extraction, the list was reviewed collaboratively by the research team and refined iteratively to support capture of all cognition-related words and phrases. Where additional terms emerged during extraction, these were brought back to the team for discussion. Terms were then later grouped into categories of similar meaning for reporting through team discussion and consensus. If the same term appeared multiple times within a single chart during the 24-hour review period, each instance was counted.

This study was conducted by an interdisciplinary team of clinicians and academics as part of a health directorate vacation scholar program. Data extraction was undertaken by a nursing student nearing completion of their degree, who had a research interest in delirium detection in hospital settings. The student received preparation from the research team and was closely supervised by an associate professor of nursing throughout the study. The wider research team included hospital-based clinicians and academic researchers with expertise in cognitive care, digital health records, and chart audit methodology (Bail et al., 2021, 2023; Connolly et al., 2025). Regular meetings were held during the data extraction phase to discuss interpretation, resolve uncertainties, and support consistency in collection and recording.

### 2.4 Ethical considerations

Ethical approval was granted prior to conducting this research by the Australian Capital Territory (ACT) Health Human Research Ethics Committee Low Risk Sub-Committee (ACT reference number 2021.LRE.00222; Research Ethics and Governance Information System [REGIS] reference number 2021/ETH12372). As this study was a retrospective audit of existing records, there was no direct patient contact and ethical approval covered secure handling and de-identification of collected data; this study was, therefore, not registered.

## 3 Results

Of 807 inpatient clinical records identified at the midnight census, 460 were eligible for inclusion in this study. The mean age of patients was 67.4 years (range 16–101), 221/460 (48%) were female, and 12/460 (3%) identified as Aboriginal or Torres Strait Islander. The mean length of hospital stay was 27 days (range 2–166), and most patients were admitted under the specialty of geriatrics.

### 3.1 Screening with the 4AT

**Fig. 2:**
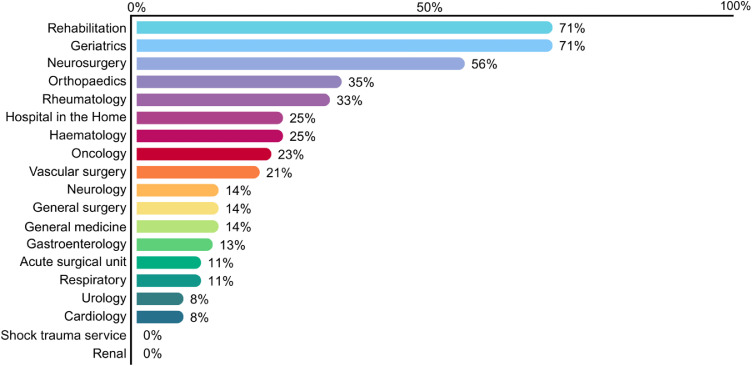
Percentage of at-risk inpatients who received screening with 4AT by admitting specialty.

Delirium risk factors were documented in 316/460 (69%) records, with age ≥65 years being the most identified risk factor (293/316; 93%). All 316 at-risk patients were expected to receive 4AT screening within 24 hours of risk factor identification, as per local hospital policy. A 4AT was completed within 24 hours for 28/316 (9%) patients, and after 24 hours for 80/316 (25%) patients; totalling 108/316 (34%) at-risk patients who were screened with a 4AT (Table 1). Across the admitting specialties, the percentage of at-risk patients who received a 4AT varied (range 0–71%), with rehabilitation and geriatrics both having the highest screening rates (Fig. 2).

**Table 1:** Delirium care pathway documentation among at-risk inpatients (n=316).

Delirium Care Pathway Stage	*n* (%)
**Patients with risk factors identified**	316 (100)
**Screening**	
4AT completed (any time)	108 (34)
4AT within 24 hrs	28 (9)
4AT score 1–3 (possible cognitive impairment)^a^	59 (19)
4AT score ≥4 (possible delirium)^b^	25 (8)
**Assessment and Diagnosis**	
CAM completed	12 (4)
Delirium documented (CAM/medical officer)	3 (1)

*Abbreviation:* CAM=Confusion Assessment Method.

a Indicates possible cognitive impairment and need for further assessment as per local hospital policy.

b Suggests possible delirium but is not diagnostic.

**Exclusions:** Specialties with <6 patients and patients without a recorded admitting specialty are not included

### 3.2 4AT scores and indication for further assessment

Among the at-risk patients, 59/316 (19%) had a 4AT score between one and three (indicating possible cognitive impairment and a need for further investigation), and 25/316 (8%) had a 4AT score of four or more (indicating possible delirium but is not diagnostic), totalling 84/316 (27%) of patients who had scores indicating the need for further assessment as dictated by local hospital policy. A Confusion Assessment Method was documented for 12/316 (4%) patients, and delirium was recorded as a diagnosis in 3/316 (1%) at-risk patients (Table 1).

### 3.3 Language in documentation 24 hours following screening

In the 108 records where a 4AT screening was conducted, documentation over the subsequent 24 hours contained observations of cognition in 106/108 (99%) records. Across the records, 331 words and phrases related to cognition were collected, counted, and categorised (Table 2). The most frequent categories were *alertness and orientation* (129/331; 39%) and *confusion* (72/331; 22%). References to *level of consciousness* made up 34/331 (10%) of words and phrases. Mentions of standardised screening or assessment accounted for 16/331 (5%) of the words and phrases, and, within these, the Glasgow Coma Scale was mentioned more than twice as often as the 4AT. Diagnostic terms (including ‘delirium,’ ‘dementia,’ ‘ongoing progressive cognitive decline,’ ‘cognitive impairment’) comprised 11/331 (3%) of all terms. The word ‘delirium’ appeared six times (2% of all words). The remaining categories included descriptions of mood and behaviour (18%) (Table 2).

**Table 2:** Cognitive language documented in clinical record 24 hours after 4AT screening (331 terms from 106 records).

Words and Phrases	Count	Category	Total	%
Alert and orientated	68	Alert and orientated	129	39
Alert	52			
Orientated to time, place, and person	6			
Nil confusion	2			
Mented	1			
Confused	39	Confusion	72	22
Pleasantly confused	18			
Not orientated to time, place, or person	13			
Slowed mentation	1			
Unpleasantly confused	1			
Awake	25	Level of consciousness	34	10
Drowsy/Sleepy	6			
Incomprehensible sounds	2			
Asleep	1			
Agitated	10	Descriptors of negative mood	25	8
Restless	6			
Compulsive/impulsive	5			
Hallucinations/paranoia	2			
Wandering	1			
Anxious	1			
Communicates well/interactive	4	Settled/interactive behaviour	17	5
Pleasant	4			
Resting in bed	4			
Settled	3			
Nods and smiles	1			
Talkative	1			
Redirectable	4	Able to follow instructions/redirect	16	5
Cooperative	4			
Obeying	3			
Responsive	3			
Reorientate	2			
GCS (Glasgow Coma Scale)	11	Standardised screening/assessment	16	5
4AT	5			
Disruptive	4	Indicators/descriptors of negative behaviours	11	3
Aggressive	4			
Swearing	1			
Non-compliant	1			
Limb restraints	1			
Delirium	6	Diagnostic language	11	3
Ongoing progressive cognitive decline	3			
Dementia	1			
Cognitive impairment	1			

## 4 Discussion

**Fig. 3:**
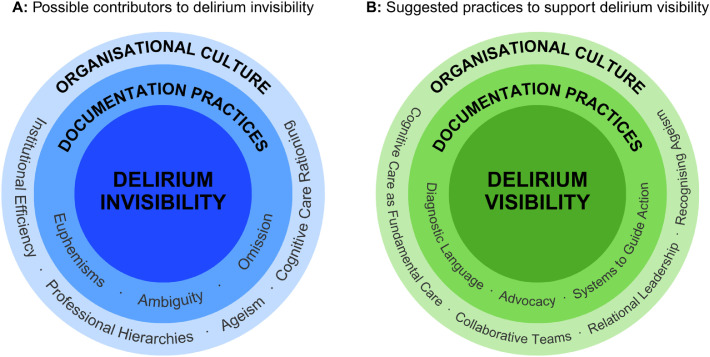
Conceptual model of possible contributors to delirium invisibility and suggested practices to support delirium visibility in hospital care.

In this hospital-wide study, delirium risk factors were commonly documented for 69% (316/460) of inpatients. However, subsequent screening and assessment was inconsistent. Only one third (34%) of at-risk patients received 4AT screening, and only 9% were screened within 24 hours. This finding is consistent with recent international evidence of low delirium screening rates, reported at 27.3% by the Geriatric Medicine Research Collaborative (2022) and 44.5% by Codling et al. (2021). Additionally, only 4% (12/316) of inpatients were assessed using the Confusion Assessment Method, and only 1% (3/316) had delirium documented as a diagnosis. Screening completion of at-risk inpatients was high in geriatrics (71%) and rehabilitation (71%) specialties, but low in general surgery (14%) and the acute surgical unit (11%). Overall, these findings illustrate substantial attrition at each step in the delirium care pathway, from risk identification to screening to diagnostic assessment (Table 1) and substantial variation in documented screening by admitting specialty (Fig. 2). As this was a clinical record audit, these findings reflect documented practice rather than all care that may have been provided. Nevertheless, the patterns in the findings suggest missed opportunities for documented recognition and escalation of delirium. Fig. 3 and the following section synthesises these findings and the possible implications for practice.

Panel A illustrates possible contributors to delirium invisibility in hospital care, informed by the documentation patterns observed in this study and interpreted in relation to relevant literature. These include documentation practices such as euphemism, ambiguity, and omission, alongside broader organisational culture including institutional efficiency, professional hierarchies, ageism, and cognitive care rationing. Panel B presents suggested practices that may support delirium visibility, including documentation practices using diagnostic or pre‑diagnostic language, advocacy, and systems to guide action, alongside organisational culture that may support delirium recognition and response, such as cognitive care as fundamental care, collaborative teams, relational leadership, and recognising ageism. Together, the panels depict how shifts in documentation practices and organisational culture may support progression along delirium care pathways. This figure is a conceptual interpretation informed by the findings and relevant literature; it is not an empirically tested or explanatory model.

### 4.1 Ambiguity, euphemisms, and omission in documentation

We identified that cognitive observations were frequently documented in the 24 hours following screening (106/108; 99%), yet delirium itself was rarely named. Across 331 documented words and phrases, *diagnostic terminology* accounted for only 11/331 (3%), with the word ‘delirium’ appearing only six times (2%) (Table 2). Documentation language was dominated by ambiguous descriptions of cognition. Words and phrases related to *confusion* accounted for 72/331 (22%) of terms, with ‘confused’ (39/72; 54%) and ‘pleasantly confused’ (18/72; 25%) accounting for most of these. Language referring to *alertness and orientation* accounted for 129/331 (39%) of the documented words and phrases, including ‘alert and orientated’.

From the findings, we suggest that cognitive change was often observed and described in the clinical record, but that further action was not documented. Previous researchers have confirmed that the term ‘delirium’ is often omitted from documentation, and ambiguous descriptors, such as ‘confused’ or ‘disoriented,’ continue to be used by healthcare practitioners in documentation, even when delirium is confirmed (Puelle et al., 2015; Welch and Jackson, 2018). In practice, omission of diagnostic language (‘delirium’) reduces the visibility of delirium in clinical communication, including handovers and clinical record coding (using International Classification of Diseases), and contributes to the persistent under-recognition and under-reporting of delirium (Bowman et al., 2024; Casey et al., 2019; Connolly et al., 2025). Euphemistic phrasing, such as ‘pleasantly confused,’ may also soften the perceived urgency of cognitive deterioration and disguises a possible delirium. Although ‘pleasantly confused’ is familiar in practice, it has received no attention in the literature. In the context of this study, such language may reflect a way of documenting cognitive change that signals no further need for assessment or escalation and a possible explanation as to why delirium care pathway progression fails.

### 4.2 Delirium care pathways and professional roles

Within this study, local hospital policy required a medical officer to complete further assessment with a Confusion Assessment Method when screening indicated possible cognitive impairment. Documented completion of the Confusion Assessment Method was low (12/316; 4%), despite frequent risk factor identification (316/460; 69%) and some documented screening (108/316; 34%). One possible explanation is that progression along the delirium care pathway may be affected by role delineation within local policy and workflow. It is possible the division of responsibilities created a dependency at the assessment step in the pathway, where nurses were able to identify risk and undertake screening, but further diagnostic assessment relied on medical review.

Professional roles and hierarchies can influence how delirium is recognised, reported, escalated, and acted on in practice. Puelle et al. (2015), for example, found that nurses more commonly used more observational terms, such as ‘confused’ or ‘disoriented,’ whereas medical officers used more diagnostic terms, such as ‘delirium’ or ‘altered mental status.’ Within this study, however, diagnostic language was scarce across documentation overall, accounting for only 3% (11/331) of the documented words and phrases. Nurses are often well positioned to notice cognitive changes early or to hear concerns raised by families and care partners, which is vital for timely detection and management of delirium (Aggar et al., 2022). Researchers have described how nurses can feel unheard or dismissed when escalating cognition and delirium concerns to medical officers (Ragheb et al., 2023), and Bail et al. (2009) have described how hospital policy and role expectations can constrain nursing agency in practice. Therefore, documented practice may be shaped by policy expectations, interdisciplinary workflows, and the level of authority practitioners perceive they have to escalate cognition concerns. Although this audit could not examine these factors, they may help explain the attrition observed in documentation along the delirium care pathway. We recommend further exploration in qualitative or observational research as to how delirium concerns are communicated and acted upon in hospital settings is needed.

### 4.3 Specialty variation and delirium risk identification

Patients with a documented risk factor for delirium were common in this study (316/460; 69%), with being aged ≥65 years the most frequently identified risk factor. Geriatric and rehabilitation specialties achieved the highest documented screening rates for at-risk patients (71%), while surgical specialties were much lower, despite surgery being a recognised risk factor in local hospital policy (general surgery 14%; acute surgical unit 11%) (Fig. 2). We interpreted these findings to indicate that delirium risk may be recognised differently across specialties and clinical contexts. One possible explanation is that delirium screening competes with other priorities in acute care environments, particularly where institutional efficiency and patient flow are performance measures. Researchers have linked these institutional efficiency pressures with missed care and the rationing of fundamental care, including cognitive screening and assessment (Bail and Grealish, 2016; Connolly et al., 2025; Grealish et al., 2023). In this context, lower documented screening rates may reflect the priority given to cognition within different specialities and care settings.

A further possible explanation is that broader assumptions about age and cognition shape how delirium risk is interpreted. Even though older people are disproportionately affected by delirium, national standards mandate early risk identification and screening across all patients and specialties because delirium is known to be missed in certain groups, including those with hypoactive presentations, females, and those aged under 60 years (Australian Commission on Safety and Quality in Health Care, 2021b; Jäckel et al., 2021). Healthcare practitioners’ views of older people, and ageism more broadly, may also normalise confusion as an expected part of ageing, leading to a lack of investigation and action on cognitive changes (Australian Human Rights Commission, 2025; Henry et al., 2024; Mazzola and Hascup, 2025). Understood within this context, the phrase ‘pleasantly confused’ identified in this study may reflect a tendency to soften or normalise cognitive impairment, rather than communicate urgent assessment and intervention. From this audit, we cannot establish a link between ageism and institutional efficiency and the documentation patterns, but these concepts potentially provide a useful lens to warrant further investigation in future studies.

### 4.4 Use of standardised tools in practice

The use of validated screening and assessment tools was mandated in local hospital policy in this study, yet only 9% of at-risk patients received documented 4AT screening within 24 hours, and only 4% had a documented Confusion Assessment Method completed. Within the documentation language audited, the standardised screening and assessment grouping accounted for 16/331 (5%) words and phrases. Glasgow Coma Scale scores were noted more than twice as often (11/16; 69%) as 4AT screening (5/16; 31%). This pattern may suggest that embedded neurological assessment pathways were documented more routinely than cognitive screening practices.

Workload pressures experienced by healthcare practitioners may be a possible explanation for the low use of delirium screening and assessment tools found in this study. Redley and Raggatt (2017) identified that the proliferation of risk and assessment forms in hospitals is burdensome and can make it difficult for healthcare practitioners to determine what to prioritise (Redley and Raggatt, 2017). Further, practitioners may rely on more familiar and task-based care when experiencing workload and system pressures while caring for multiple patients (Bail and Grealish, 2016; Lee and Roh, 2023). These pressures may also cause healthcare practitioners to confuse neurological and cognitive assessment tools, viewing the Glasgow Coma Scale and 4AT as duplication. Education on delirium tools and guidelines remains necessary, but education in isolation is unlikely to be sufficient (Cody et al., 2021; Teodorczuk and Billet, 2017). Increasing delirium screening and assessment using validated tools in practice may require attention on workflow and documentation systems that support their routine use, alongside education strategies.

### 4.5 Implications for practice

We have highlighted several steps in the documented delirium care pathway where recognition and response failed. To support movement in delirium practice from invisibility to visibility and action, several practice implications could be considered. First, clearer and more intentional documentation language may improve delirium visibility in the clinical records. In this study, cognition was commonly observed and described, yet diagnostic language was scarce. Terms such as ‘confused’ and ‘pleasantly confused’ acknowledge altered cognition but may not communicate urgency or support patient advocacy as clearly as diagnostic or pre-diagnostic language. Documenting ‘possible delirium’ may provide a clear cue for early recognition and an ethical imperative to act (Lin et al., 2023), including considering initiation of non-pharmacological interventions. When screening results, observations, or family members raise concern about possible delirium, clearer diagnostic language in the clinical record may improve delirium visibility and assist in further assessment, inclusion in clinical handover, and delirium care pathway progression.

Second, we suggest that progression from screening to assessment may be strengthened by reviewing how delirium care pathways are embedded within workflow practices. Documentation prompts, automated escalation pathways, and alerts linked to identified risk factors may support delirium being more visible in routine practice. An existing comparable example in Australia is the SEPSIS KILLS program, which links vital sign monitoring, clinical tools, escalation pathways, and policy with time sensitive steps for action by practitioners (Burrell et al., 2016; Clinical Excellence Commission, n.d.). Although delirium differs from sepsis, both are time critical and varied in presentation, require interdisciplinary intervention, and may have long-lasting or fatal outcomes for patients. This comparison may be useful when considering system-level approaches to improving delirium visibility and action.

Finally, delirium education initiatives remain important but are likely to be most effective when paired with broader system support. Researchers have demonstrated that educational interventions can improve screening and detection of delirium and contributes to changes in practice culture (Benn et al., 2025; Cody et al., 2021; Lee et al., 2020; Mudge et al., 2013; Murray et al., 2019). However, we suggest that documentation practices may be influenced by broader organisational culture, workflow, and workload pressures. Improving delirium practice may benefit from leadership that values cognitive care as essential to patient safety and fosters environments where practitioners feel safe to escalate concerns (Grealish et al., 2023). Reimagining delirium practice may, therefore, involve attention to healthcare practitioner knowledge, as well as the organisational culture that influences how cognition is recognised, documented, and acted upon. These implications are brought together in Fig. 3, which conceptually depicts possible contributors to delirium invisibility and suggested practices to support delirium visibility in hospital practice.

### 4.6 Limitations

We conducted this study at a single site. It was a retrospective audit of routinely collected data in clinical records, and, therefore, the results reflect what was documented and not necessarily all care that was provided. The point-prevalence design provided a snapshot of practices related to the screening, assessment, and documentation of delirium; therefore, we cannot imply or predict trends over time. Additionally, the 24-hour timeframe utilised to capture cognitive language in documentation may not capture all documentation related to cognition or delirium across an inpatient length of stay. The extraction and categorisation of words and phrases related to cognition were supported through piloting, supervision, and regular team discussion and consensus, but formal inter-rater reliability testing or duplicate independent coding were not undertaken. The audit was conducted in July 2021 and reflects documentation practices at that timepoint rather than current practice; however, these findings are likely to still be relevant as the under-recognition and inconsistent documentation in hospitals about delirium continues to be reported in recent literature.

Data collection occurred during the COVID-19 pandemic. Although the study site was at a hospital in Canberra, Australia, where pandemic-related disruption was less severe than in many other national and international locations, changes to staffing and clinical priorities during this period may have influenced documentation practices and should be considered when interpreting the findings and their external validity. While this was a single site study, the hospital operated under national standards (Comprehensive Care Plan and the Australian Delirium Clinical Care Standard), and, therefore, the processes and structures may be comparable to other Australian acute care hospitals. The findings of this study, therefore, may be transferable to similar settings where standardised delirium care pathways are in place.

## 5 Conclusions

We found that, in this hospital, inpatients at-risk of delirium were identified by healthcare practitioners; however, subsequent screening, assessment, and diagnostic documentation were scarce. Additionally, language used to describe cognitive observations in clinical record documentation was ambiguous, with terms such as ‘alert and orientated’ and ‘pleasantly confused’ used in preference to diagnostic terms (such as ‘delirium’). These documentation practices may reduce the visibility of delirium and suggest missed opportunities for documented recognition and escalation of delirium in hospital practice. We recommend healthcare practitioners consider the use of diagnostic or pre-diagnostic language in clinical record documentation and approaches that support action on possible delirium within their scope of practice. Educational programs remain important to increase delirium knowledge and skills amongst healthcare practitioners but may be most effective when paired with organisational culture and systems that support cognitive care in routine hospital practice.

## Funding

Eleanor Brace was supported to undertake this research through a Vacation Scholarship program operated by the Australian Capital Territory (ACT) Health Directorate, sponsored by Anthony Dombkins, Chief Nursing and Midwifery Officer of ACT Health.

## Ethics approval statement

Ethical approval was granted prior to conducting the research by the Australian Capital Territory (ACT) Health Human Research Ethics Committee Low Risk Sub-Committee (ACT reference number 2021.LRE.00222; Research Ethics and Governance Information System [REGIS] reference number 2021/ETH12372).

## CRediT authorship contribution statement

**Kelly Marriott-Statham:** Writing – review & editing, Writing – original draft, Visualization, Methodology, Conceptualization. **Eleanor Brace:** Writing – review & editing, Visualization, Validation, Methodology, Investigation, Data curation. **Seán Hambrook:** Writing – review & editing, Validation, Supervision, Resources, Methodology, Funding acquisition, Conceptualization. **Chrysta Bridge:** Writing – review & editing, Validation, Supervision, Resources, Methodology, Funding acquisition, Data curation, Conceptualization. **Kasia Bail:** Writing – review & editing, Visualization, Validation, Supervision, Resources, Project administration, Methodology, Funding acquisition, Formal analysis, Data curation, Conceptualization.

## Declaration of competing interest

The authors declare that they have no known competing financial interests or personal relationships that could have appeared to influence the work reported in this paper.

## Data Availability

Further data that support the findings of this study are available from the corresponding author upon reasonable request.

## Appendix Supplementary materials

Image, application 1
